# Factors associated with patient satisfaction in laparoscopic adrenalectomy

**DOI:** 10.1016/j.heliyon.2019.e01909

**Published:** 2019-06-15

**Authors:** Jakrapan Wittayapairoch, Suriya Punchai, Kamonwan Jenwitheesuk, Verajit Chotmongkol, Kittisak Sawanyawisuth, Kriangsak Jenwitheesuk

**Affiliations:** a Department of Surgery, Thailand; b Department of Medicine, Faculty of Medicine, Thailand; c North-eastern Stroke Research Group, Sleep Apnea Research Group, Research Center in Back, Neck and Other Joint Pain and Human Performance, Research and Training Center for Enhancing Quality of Life of Working Age People, and Research and Diagnostic Center for Emerging Infectious Diseases (RCEID), Khon Kaen University, Khon Kaen, Thailand

**Keywords:** Surgery, Internal medicine, Medicine, Headache, Age, Predictors, Conn's syndrome

## Abstract

Laparoscopic adrenalectomy a treatment that is recommended for patients with adrenal adenoma and has been shown to lead to a 94% biochemical remission rate of aldosterone as well as improvements to quality of life in five domains of the SF-36. This method is also associated with high rates of patient satisfaction. However, there is little information available on the factors associated with patient satisfaction in cases of laparoscopic adrenalectomy. This study aimed to evaluate these factors in patients with Conn's syndrome who underwent laparoscopic adrenalectomy. This study was based on a survey and was conducted at Srinagarind Hospital at the Khon Kaen University Faculty of Medicine in Thailand. The inclusion criteria were that patients were between 15 and 60 years of age, had been diagnosed with adrenal gland tumors, and had undergone trnasperitoneal laparoscopic adrenalectomy. All eligible patients were asked to fill out a self-report questionnaire in which they rated their satisfaction (out of 10) and factors associated with their level of satisfaction in the areas of clinical treatment and scarring. There were 44 patients who participated in the study. The average (SD) age of all patients was 47.10 (10.90) years. The average overall satisfaction scores for the surgery and with regard to scarring post surgery were 9.47 (1.15) and 8.11 (2.21), respectively. Only the presence of headaches was an independent factor associated with the overall satisfaction, with a coefficient of -0.29 (p value 0.001). Only age was significantly predictive of overall satisfaction with regard to scarring with a coefficient of 0.05 and p value of 0.046. In conclusion, the presence of headaches was related to overall satisfaction and age was associated with satisfaction with regard to scarring in patients Conn's syndrome who underwent laparoscopic adrenalectomy.

## 1 Introduction

Conn's syndrome is a treatable type of secondary hypertension. Transperitoneal laparoscopic adrenalectomy has been shown to be a better treatment option than open surgery in these cases, as it requires a smaller incision [1]. The conversion rate to open surgery in cases of laparoscopic adrenalectomy has been reported to be 4.2% (out of 215 cases) over 10 years. However, none of these cases involved patients with Conn's syndrome [2]. Laparoscopic adrenalectomy can be performed using a needlescopic technique in cases in which the tumor is under 5 cm in diameter or is in a single site [3, 4]. The biochemical remission rate of aldosterone has been shown to be 94%, with 37% of cases resulting in complete clinical remission after laparoscopic adrenalectomy [5]. The treatment has also been shown to significantly improve quality of life in five domains of the SF-36 including physical health, general health, emotional health, mental health, and vitality [6].

Laparoscopic adrenalectomy has also been shown to result in shorter hospital stays (three vs five days), fewer patients being administered narcotics (28 vs 48), and faster return to normal status (3.8 vs seven weeks) compared with open posterior adrenalectomy, all with p values of less than 0.05. Average patient satisfaction scores have also been shown to be significantly higher in cases of laparoscopic surgery than in open surgery (9 vs 7) [7]. In addition, several studies have found that patients prefer laparoscopic adrenalectomy to open surgery [4, 8]. There is little information available on the factors associated with patient satisfaction in cases of laparoscopic adrenalectomy. This study aimed to evaluate the factors related to high satisfaction scores in patients with Conn's syndrome who underwent laparoscopic adrenalectomy.

## 2 Methods

This study was based on a survey and was conducted at Srinagarind Hospital at the Khon Kaen University Faculty of Medicine in Thailand. The inclusion criteria were age between 15 and 60 years, being diagnosed with an adrenal gland tumor, and having undergone transperitoneal laparoscopic adrenalectomy. Patients who were pregnant or immunocompromised were excluded. The study period was between 2011 and 2017.

All eligible patients were asked to fill out a self-report questionnaire. The questionnaire included questions about the patient's age, sex, clinical symptoms, history of hypertension, history of taking antihypertensive medications (both before and after surgery), scarring, and overall satisfaction with the laparoscopic adrenalectomy and with regard to scarring.

Patients' clinical symptoms included problems with movement, self-care, and engaging in daily activities, as well as pain/fatigue, anxiety/depression, weakness, potassium replacement, abdominal symptoms, previous history of intestinal obstruction, and headache/dizziness [9]. Scarring was rated in terms of five characteristics: pain, itchiness, color, induration, and hardness. Patient's scores with regard to headache, scarring, scar characteristics, and overall satisfaction with the surgery ranged from zero to 10 with the best score being 10.

Statistical analysis. Descriptive statistics were used to calculate averages (SD) or numbers (percentage). Factors associated with overall satisfaction in the areas of surgery and scarring were determined using multivariate linear regression analysis. Factors with p values of less than 0.20 were considered clinically significant and were included in the final model for multivariate linear regression analysis. The final model was used to determine the coefficient, standard error, and p value of each factor. The adjusted R squared of the final model was also reported. All statistical analysis was performed using STATA software (College Station, Texas, USA).

Ethical consideration. The study protocol was approved by the ethic committee in human research, Khon Kaen University, Thailand (HE611074). An informed consent from eligible patients was given prior to the study participation.

## 3 Results

There were 44 patients who participated the study, all of whom had adrenal adenoma, with an average (SD) age of 47.10 (10.90) years (Table 1). Male patients accounted for 27.91% of participants and abdominal symptoms were the most common symptom (15 patients; 34.09%). The average (SD) headache score was 1.20 (2.35) and 38.64% of patients were taking antihypertensive medications. The overall satisfaction scores with regard to surgical scarring were 9.47 (1.15) and 8.11 (2.21), respectively, as shown in Table 1.

**Table 1:** Characteristics of studied variables in patients with adrenal gland tumors who underwent transperitoneal laparoscopic adrenalectomy (n = 44).

Factors	Mean (SD) or number (percentage)
Age, years	47.10 (10.90)
Male sex	12 (27.91)
Movement problems	3 (6.82)
Self-care problems	0
Daily activity problems	7 (15.91)
Pain or fatigue	11 (25.00)
Anxiety/depression	
No	38 (86.36)
Mild	4 (9.09)
Moderate	2 (4.55)
Weakness	2 (4.55)
Potassium replacement	1 (2.27)
Abdominal bloating/dyspepsia/constipation	15 (34.09)
Previous history of intestinal obstruction	1 (2.27)
Number of patient with headache	11 (25.00)
Headache score*	1.20 (2.35)
Taking antihypertensive medications	17 (38.64)
Numbers of antihypertensive medications	
No	27 (62.79)
Same as before surgery	8 (18.60)
Fewer than before surgery	8 (18.60)
Scar characteristics, score*	
Pain	0.34 (0.88)
Itchiness	0.19 (0.50)
Color	3.50 (2.96)
Induration	1.53 (2.63)
Hardness	1.25 (2.09)
Overall satisfaction of scar	8.11 (2.21)
Overall satisfaction*	9.47 (1.15)

Note. *range 0–10.

There were seven factors in the final model that predicted overall satisfaction (Table 2). Only the presence of headaches was an independent factor associated with overall satisfaction with a coefficient of -0.29 (p value 0.001). The adjusted R squared for the final model was 0.32. The overall satisfaction score of patients with headache was significantly lower than those without headache (8.81 ± 1.53 vs 9.69 ± 0.91; p value 0.011). Only age significantly affected satisfaction with regard to scarring in the final model, with a coefficient of 0.05, p value of 0.046 (Table 3), and an adjusted R squared of 0.16.

**Table 2:** Factors associated with overall satisfaction in patients with adrenal gland tumors who underwent transperitoneal laparoscopic adrenalectomy (n = 44).

Factors	Coefficient	Standard error	p value
Age	0.01	0.01	0.429
Sex	0.18	0.38	0.631
Headache	-0.29	0.08	0.001
Weakness	1.07	0.79	0.188
Anxiety/depression	0.68	0.37	0.075
Potassium replacement	0.31	1.21	0.798
Scar satisfaction	-0.16	0.10	0.102

**Table 3:** Factors associated with overall scar satisfaction in patients with adrenal gland tumors who underwent transperitoneal laparoscopic adrenalectomy (n = 44).

Factors	Coefficient	Standard error	p value
Age	0.05	0.02	0.046
Headache	-0.25	0.12	0.054
Scar color	-0.15	0.11	0.204
Scar induration	-0.16	0.13	0.242
Scar hardness	-0.15	0.15	0.309

## 4 Discussion

We found that overall patient satisfaction with laparoscopic adrenalectomy was high (9.47/10), which is similar to the results of previous studies [3, 4, 7]. One previous study, for example, reported an average score of 9/10 on the visual analogue scale for satisfaction [7]. This study also found patient satisfaction with regard to surgical scarring from laparoscopic adrenalectomy to be high (8.11/10) [1].

Although a previous study found that patients’ scores were higher when they were left with smaller scars [1], in our study, only headache was negatively associated with overall patient satisfaction (Table 2). If patients suffered from headaches after laparoscopic adrenalectomy, the satisfaction scores were an average of 0.29 lower after adjustment for various other factors, as shown in Table 1. This was also true of their satisfaction scores with regard to scarring.

A previous study found that patients with unilateral primary aldosteronism had almost 100% biochemical remission, but that blood pressure control was successful in only 47% of patients [5]. Similarly, in our study, only eight out of 17 patients were administered fewer antihypertensive medications after laparoscopic adrenalectomy (47.05%; Table 1). Hypertension may cause headaches, particularly in cases of hypertensive crises [10]. In addition, a previous study found that hypertensive crises was caused by hyperaldosteronism in 14.3% of cases, which may lead to poor quality of life and emergency room visits [11]. We, therefore, hypothesize that effective control of hypertension was a crucial factor in overall patient satisfaction in the patients in our study with Conn's syndrome who underwent laparoscopic adrenalectomy.

In our study, headache was not significantly associated with patient satisfaction with regard to scarring (Table 3). Age, however, was positively correlated with patients’ satisfaction scores in this area (coefficient of 0.05; p value of 0.046), as shown in Table 3. Older patients tended to be have higher satisfaction scores in the area of surgical scarring from laparoscopic adrenalectomy. A previous report also found that age was associated with satisfaction in this area, particularly in cases in which patients were left with only a small scar [12]. Patients under 50 years old who underwent single site laparoscopic adrenalectomy had significantly higher satisfaction scores than those who underwent conventional laparoscopic adrenalectomy (9.17 vs 6.38; p 0.036).

There were some limitations to this study. First, the study population included only Thai patients with Conn's syndrome. Second, there was no comparison or randomization implemented in this study. However, recent studies showed that laparoscopic adrenalectomy is safe and may be considered as a standard treatment or equal to open adrenalectomy. The benefits of laparoscopic adrenalectomy over the open method included short hospital stay, less pain, and better cosmetic outcome [13, 14]. Finally, the study used a small sample size. Although it was able to show significant factors for both overall satisfaction and satisfaction in the area of scarring, future studies with larger sample sizes that include patients of other ethnicities may be needed.

In conclusion, headache was related to overall satisfaction, while age was associated with satisfaction with regard to scarring in patients with Conn's syndrome who underwent laparoscopic adrenalectomy.

## Declarations

### Author contribution statement

Jakrapan Wittayapairoch: Conceived and designed the experiments; Performed the experiments; Analyzed and interpreted the data; Wrote the paper.

Suriya Punchai, Kamonwan Jenwitheesuk: Conceived and designed the experiments; Performed the experiments; Analyzed and interpreted the data.

Verajit Chotmongkol, Kittisak Sawanyawisuth: Analyzed and interpreted the data; Wrote the paper.

Kriangsak Jenwitheesuk: Conceived and designed the experiments; Performed the experiments; Analyzed and interpreted the data; Contributed reagents, materials, analysis tools or data; Wrote the paper.

### Funding statement

This research did not receive any specific grant from funding agencies in the public, commercial, or not-for-profit sectors.

### Competing interest statement

The authors declare no conflict of interest.

### Additional information

No additional information is available for this paper.
